# Record of *Leptometopa latipes* (Diptera: Milichiidae) from a human cadaver in the Mediterranean area

**DOI:** 10.1080/20961790.2018.1490473

**Published:** 2018-10-09

**Authors:** Giorgia Giordani, Fabiola Tuccia, Silvia Zoppis, Carla Vecchiotti, Stefano Vanin

**Affiliations:** a School of Applied Sciences, University of Huddersfield, Huddersfield, UK;; b Dipartimento di Scienze Anatomiche Istologiche Medico Legali e dell’Apparato Locomotore, Section of Legal Medicine, Laboratory of Forensic Genetics, University of Rome "Sapienza", Rome, Italy

**Keywords:** Forensic entomology, postmortem interval, decomposition, *Leptometopa latipes*

## Abstract

In forensic entomology, insects are used mainly to obtain information about the time since death. This information is obtained studying the developmental rate of the first colonizers, principally species in the families Calliphoridae, Sarcophagidae, Muscidae, Stratiomyidae and Phoridae. However, species belonging to other families can provide information about body transfer or the season of the death. Among them Milichiidae are flies rarely reported from human cases despite the larvae of some species are known as saprophagous feeding on plant and animal decomposing matter. A potential cause of the lack of records of these species from forensic cases can be related with the paucity of descriptions and illustrations of the immature stages. In this article, the entomological samples collected from a human body found inside an apartment in a Maghreb country, in Northern Africa, is reported and *Leptometopa latipes* (Diptera: Milichiidae) is described in detail. Molecular analysis is also reported to confirm the morphological analysis.

## Introduction

Forensic entomology is a branch of forensic science in which insects are used as evidence in legal investigations relating to humans or wildlife [1]. The examination, identification and analysis of insects associated with human remains, combined with the knowledge of insect biology, distribution, phenology and ecology can provide a further level of detail in addition to medical and anthropological data in the reconstruction of events occurring close to the time of death. In particular, in forensic entomology, necrophagous insects are useful in estimating the minimum postmortem interval (mPMI), the movement of the cadaver after death (body transfer), the season of death, the presence of drugs or poisons [2] and, as reported in the last years, in the identification of the victim in cases of body removal before the investigators’ arrival [3].

Flies are typically the first insects to colonize a dead body. The species of flies involved differ from one location to another, but several studies have indicated that the primary species involved belong to a relatively small number of families: Calliphoridae, Sarcophagidae and Muscidae [2]. Later during the decomposition other species belonging to the Stratiomyidae, Fanniidae, Piophilidae, Phoridae families are often sampled from the body whereas species in the families of Syrphidae, Sphaeroceridae, Heleomyzidae, Sepsidae are only occasionally collected. Other species, in the families of Trichoceridae, Psycodidae, Milichiidae, Ulidiidae and Drosophilidae, are very rare and their presence depends on very specific seasonal, geographical or ecological contexts in addition to the decomposition stage of the body. These late species could provide a very detailed information about the perimortem events, as, for example, body transfer or concealment, despite generally they are useless for the mPMI estimation because the lack of developmental data and their unpredictable arrival on the body. Moreover, the paucity of records of these species can be related with the lack of a correct identification because no detailed identifications keys are available for the morphological identification of their immature stages and the incompleteness of the molecular information about these “secondary” species in Genbank or in BOLD [4–6].

The case here reported concerns the finding on a human cadaver in advanced decay of several specimens of *Leptometopa latipes* (Meigen, 1830), a fly species in the family of Milichiidae. A detailed description of the puparium and the adult of the species are here presented in order to provide support for the identification of specimens from forensic cases. Molecular analysis is also presented and discussed.

Milichiidae sometimes called “free loader flies” from their kleptoparasitic habits are small little black acalyptrate flies with larvae generally saprophagous, usually developing in decomposing plants and animals but also collected from dung and human excrements or organic matters accumulated inside the nest of ants or other social insects [2].

### Case description

In 2006, the corpse of an Italian young man was found inside an apartment in a Maghreb country, in Northern Africa. According to the first autopsy report, the cause of death was intoxication by carbon monoxide. The corpse was transferred to Italy, performing a second postmortem examination; however, the conditions of the remains made impossible determining the cause of death. Furthermore, the relatives asked for new research and analysis to confirm his identity. In 2013, the corpse was exhumed. Then, bone samples were collected, performing the kinship genetic tests. However, due to the poor conditions of the remains, these results were not successful on identity matching. The remains were heavily colonized by insects: large number of puparia and small flies were collected over the bone surface. Entomological samples were sampled, prepared and analysed following the standards and guidelines of the European Association for Forensic Entomology (EAFE) [7] and further analysed in order to obtain information useful for the investigation.

## Materials and methods

All the samples were initially observed using a Keyence VHX-S90BE digital microscope, equipped with Keyence VH-Z250R and VH-Z20R lens and VHX-2000 Ver. 2.2.3.2 software (Keyence, Osaka, Japan). Puparia samples were carefully cleaned in a water and soap solution and air-dried. No sonication was needed for the observation of the diagnostic characters. Scanning electron microscopy (SEM) observations were performed only on puparia. Dried cleaned specimens were mounted on stubs with conductive adhesive tape and coated with Au–Pd in an SC7620 Mini Sputter Coater (Quorum Technologies, Lewes, UK) and observed with a FEI QUANTA 650 FEG SEM (Thermo Scientific, Waltham, MA, USA). Pictures were directly digitized from the SEM. Terminology of morphological characters in the puparia description follows the most recent works on the topic [8–11].

To further confirm the morphological identification, a molecular DNA extraction from adult flies was performed. A 737 bp sequence of the cytochrome c oxidase subunit I (*COI*) mitochondrial gene was amplified and sequenced. The online system BLASTn^®^ [12] was used for species identification based on the percentage of identity with those available on the online gene banks. The obtained sequence is deposited on GenBank (accession number MH069729). Sequences of some Milichiidae species were downloaded from GenBank and BOLD and included in the analysis (Supplementary material Table S1). In order to increase the number of species in the analysis a region of only 533 bp was considered. Sequences were aligned with Clustal Omega [13]. A phylogenetic tree was built using the Neighbour Joining method on MEGA version 7.0 [14]. A bootstrap of 1 000 replicates was used for testing the robustness of the phylogenetic reconstruction. The tree was visualized with iTOL [15]. In the phylogenetic reconstructions, sequences of *Drosophilidae* species were considered as outgroup.

## Results

**Figure 1.**
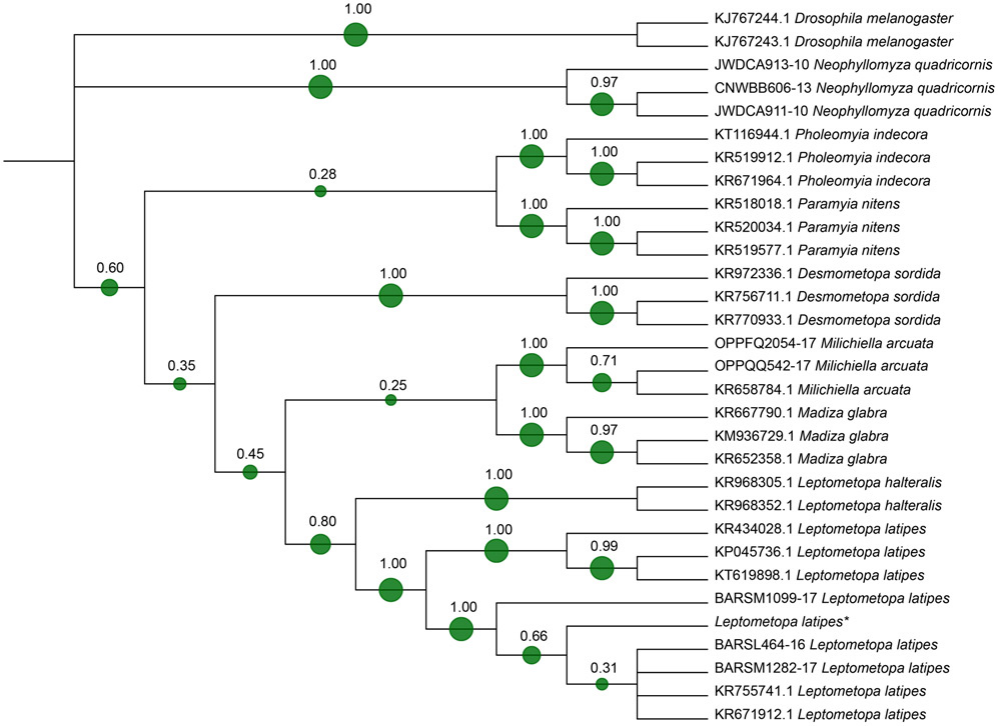
Phylogenetic tree based on Neighbour Joining method analysis of 533 bp sequence of the cytochrome c oxidase subunit I (*COI*) gene. The green spots and the number at each node indicate the bootstrap support. * indicates the sequence from this case (GenBank MH069729).

A large number of puparia and small flies were collected over the bone surfaces, identifying two different species: *Hydrotaea capensis* (Wiedemann, 1818) (Diptera: Muscidae), common species reported from decomposed and buried remains, and unexpectedly, *L. latipes* (Diptera: Milichiidae). The identification of the late species was confirmed as well by molecular analysis. The local alignment of the sequence belonging to adult flies from this case was performed using the online system BLAST^®^ (https://blast.ncbi.nlm.nih.gov/Blast.cgi). The output revealed a 99% and 97% identity, respectively, with two sequences coded in Genbank as *L. latipes* KR671912.1 and KR434028.1, with a 0.0 E value. A phylogenetic approach was used to verify the goodness of the molecular result. The tree based on 533 bp of the *COI* sequence (Figure 1) clearly confirms the morphological identification.

### Hydrotaea capensis

The old world native synanthropic species *H. capensis* was recorded from different habitats [16] and its presence is mainly reported in warm seasons. Lefebvre and Pasquerault [17] calculated for this species a minimum developmental temperature of 12.8 °C and a thermal constant expressed as Accumulated Degree Days (ADD) of (237.05±22.73) degree-days. *H. capensis* is reported to colonize exposed cadavers during the active decay stage of their decomposition [2,8] while it is also reported as one of the first colonizers in buried bodies [2,18–20].

Puparia of this species were reported from forensic and archaeological contexts all over the Europe (Portugal, Spain, France, Italy, Germany, etc.) [2,18–22]. A detailed puparium description of this species is illustrated in a forthcoming paper by Giordani et al. [8].

### Leptometopa latipes

This species has a widespread distribution in Europe, Asia, Africa and in the Nearctic and Neotropical bioregions [23–25]. Larvae belonging to this species are known to feed on decay organic matter (saprophagous) and to consume and re-digest the faeces of large animals (coprophagous) [24,25]. This species was reported from field traps and animal carrions [26,27] while, from archaeological contexts, puparia and an adult fragment of this species were reported only in the sarcophagus of Federico II in Palermo, Italy [25] (Supplementary material Table S2). In this article, we report the first record of a high number of adults and puparia (>100) in association to a human corpse.

Furthermore, because of the lack of description, we report here some illustrations and morphological description of the species focusing our attention mainly on the puparium.

**Figure 2.**
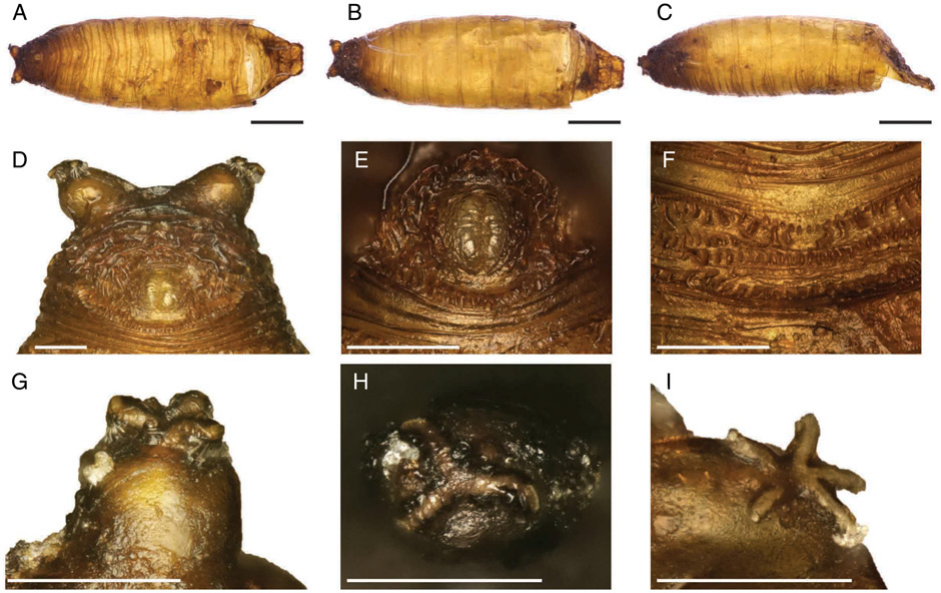
*Leptometopa latipes* puparium in ventral (A), dorsal (B) and lateral (C) view (scale bar 500 µm). Puparium details: Posterior anal region (D), anal plate (E), intersegmental spicules (F), posterior spiracle (G, H) and anterior spiracle (I) (scale bar 100 µm).

**Figure 3.**
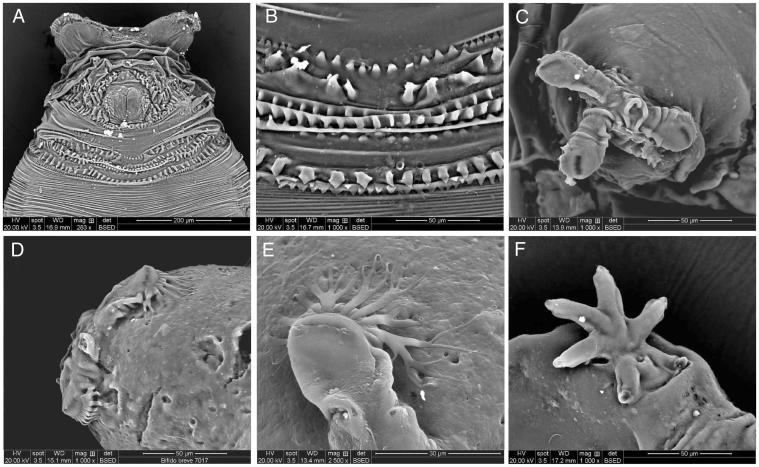
*Leptometopa latipes* puparium details: anal plate (A), intersegmental spicules (B), posterior spiracle (C, D) and filaments emanating from perispiracular glands (E), anterior spiracle (F).

Puparia of *L. latipes* are yellow to light brown (Figure 2A–C). In the analysed samples, the length of the close puparium is (0.32±0.05) mm long (*N* = 5). The posterior anal region is covered by several excrescences surrounding a smooth anal plate with no expansions (wings). All the anal papillae are absent or not discernable (Figures 2D, E and 3A). The intersegmental spines of ventral welt of abdominal segment 7 are small and differently oriented, with the top half directed towards the anal plate and the bottom half directed on the opposite direction. Spines in the external lines are smaller and closer than the central ones and ending with sharp tips (Figures 2F and 3B).

Posterior spiracles are situated on two strongly projected processes. The three slits are themselves allocated each one on a different expansion kept closed to the puparium by filaments emanating from perispiracular glands (Figures 2G, H and 3C–E). In the analysed samples, star-shape anterior spiracles showed five to six prospiracular lobes (Figures 2I and 3F).

Adults are shiny black flies and their diagnostic features are mainly associated with the frons, the thorax and the legs. In fact, *L. latipes* is a small species showing reddish margin frons, silvery microtomentose median longitudinal stripe and a bare mesopleuron. Male and female fore and mid tibia show yellow basal and medial rings. Male hind tibia is enlarged. Female body length is (1.68±0.06) mm (*N* = 3) while male body length is (1.22±0.07) mm (*N* = 5). Wings are (1.74±0.14) mm long for female and (1.31±0.25) mm long for male. On the head, the light grey ocellar triangle shows a distinctive microtomentum, two ocellar setae and three to five short central setae. The frons is rectangular, longer than wider, with anterior margin red and silvery microtomentose longitudinal stripes next to eye margin. The front presents four orbital and one lower fronto-orbital pairs of setae. Lunula is yellow and bare triangular-shaped. Antennae are dark with the first flagellomere irregularly rounded and a pubescent arista.

**Figure 4.**
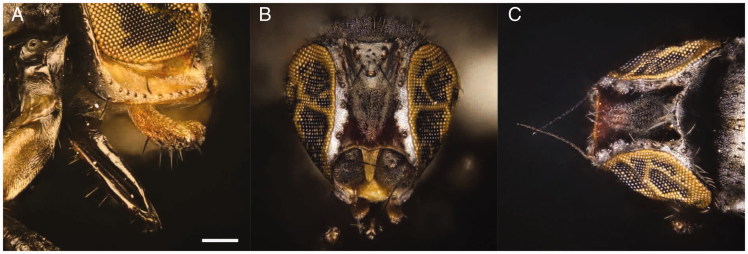
*Leptometopa latipes* adults head details. Female oral region (A), male bristles (B) and male antennae (C) (scale bar 100 µm).

Para-facial is yellow with microtomentum, gena are thick, 1/4 of eye height and yellowish. Strong vibrissae are present, located at level of lower eye margin. Palpi are yellow, slightly sickle-shaped in lateral view with black setulae in the ventral margin. The proboscis is geniculate, darkish brown, with sparse setulae in the margin (Figure 4).

**Figure 5.**
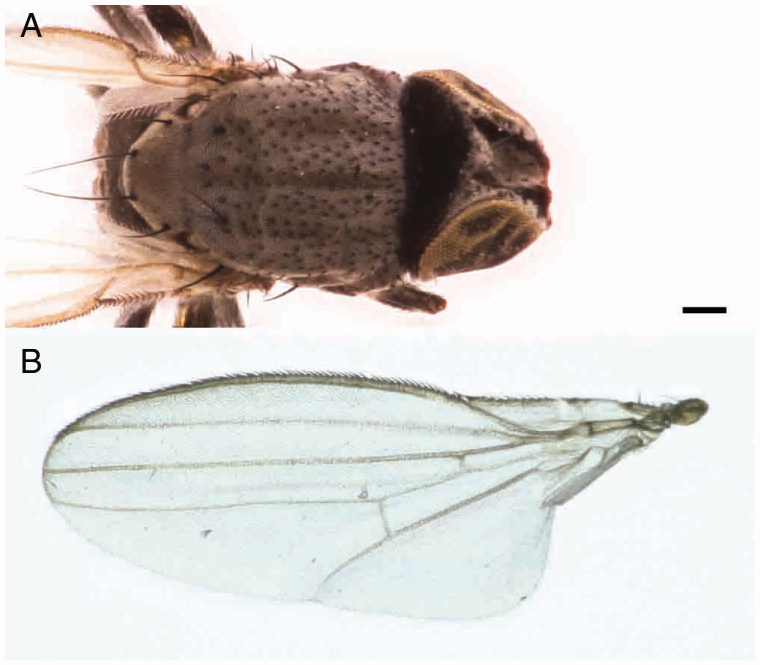
*Leptometopa latipes* adults details: thorax (A) and wing (B) (scale bar 100 µm).

**Figure 6.**
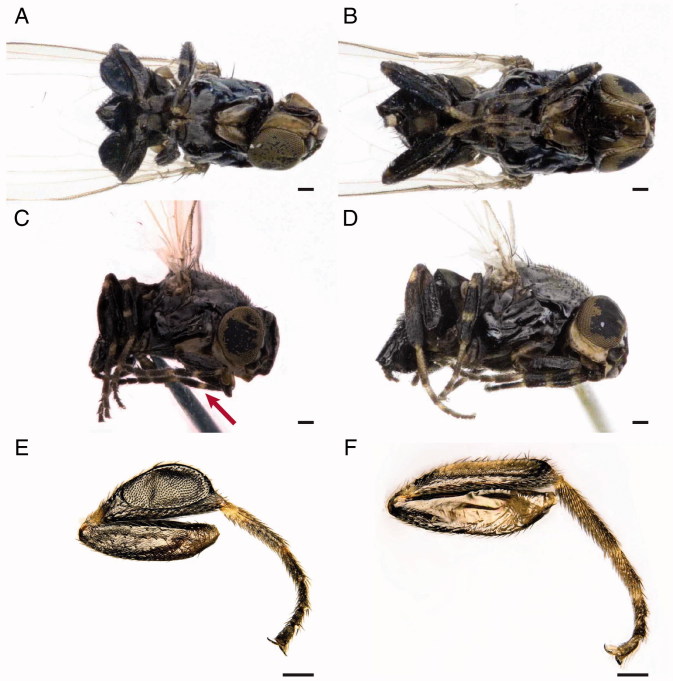
*Leptometopa latipes* adults. Male in ventral (A) and lateral (C) view. The arrow shows the yellowish ring in the foreleg. Female in ventral (B) and lateral (D) view. *L. latipes* hind tibia: male (E) and female (F) (scale bar 100 µm).

The convex thorax shows one postpronotal, one notopleural, one posterior dorsocentral, one supra- and two post-alar (in intra-alar position) pairs of setae. The scutellum presents two pairs of marginal bristles. No apical scutellar bristles are present (Figure 5A). As in other Milichiidae, the wing has two costal breaks, once near humeral cross-vein and once near apex of vein R_1_. Anal vein is extremely reduced, perceptible only as shadow (Figure 5B). A yellowish ring in the foreleg and midleg and light colour tarsi are present. Tibiae are without dorsal preapical bristle. In the male, the particular shape of the hind tibia, strongly broaden and flat, is a diagnostic character of the species (Figure 6).

## Discussion and conclusions

After death, body goes through a series of physical and biochemical transformations that create the conditions for insect colonization. The first colonizers are usually species of flies in the families Calliphoridae, Muscidae and Sarcophagidae followed by other Diptera taxa and then by Coleoptera and Lepidoptera. Hymenoptera can be present on the body as scavenger, predators or parasitoids of other insects. In the Mediterranean area, blowflies (Calliphoridae) in the genera *Calliphora*, *Chrysomya* and *Lucilia* are the most common taxa among the first exposed bodies colonizers, mainly depending on the season of the death [28,29]. In this case, the absence of blowflies can be explained with the finding of the body few hours after the death however the understanding of the time when the colonization happened in this case is very difficult because of the repeated autopsies and exhumations of the body and the initial storage in a very warm country.

Fly in the family of Muscidae are very common in body found in urban area [2,30] with some species typically found indoor as well in crypts and other hypogeal sites [31]. The Muscidae species, *H. capensis*, found on the corpse is typical of bodies that are stored in crypts or buried [2,18–20] but also it is reported from active decomposing exposed bodies [2,11]. For these reasons, its presence in this case is not surprising.

The Milichiidae species, *L. latipes*, is reported from decomposing matter, excrements and insect nests and, as other species of the same family, it is only rarely mentioned from decomposing human bodies [2]. The finding of the species in this case is very interesting and could be related to specific condition of the initial storage of the body (e.g. morgue, repeated autopsies, etc.).

From a geographical point of view both species, *H. capensis* and *L. latipes,* are present in the Mediterranean basin, a datum that is not in disagreement with the declared origin of the body, a Maghreb country. As in other cases, only an improvement in the knowledge about the species ecology and distribution would allow a better application of the entomological analysis that can complement data from other forensic disciplines like forensic pathology and anthropology.

## Supplementary Material

Supplemental Material

## References

[1] Gennard D Forensic entomology: an introduction. Chichester (UK): Wiley; 2007.

[2] Smith KGV A manual of forensic entomology. Ithaca (NY): Cornell University Press; 1986.

[3] Vanin S Advances in forensic entomology in missing persons investigations In: Morewitz SJ, Sturdy Colls C, editors. Handbook of missing persons. Cham (Switzerland): Springer International Publishing; 2016 p. 309–317.

[4] Tuccia F, Giordani G, Vanin S A general review of the most common *COI* primers for Calliphoridae identification in forensic entomology. Forensic Sci Int Genet. 2016;24:e9–e11. 27444889 10.1016/j.fsigen.2016.07.003

[5] Tuccia F, Giordani G, Vanin S A combined protocol for identification of maggots of forensic interest. Sci Justice. 2016;56:264–268. 27320399 10.1016/j.scijus.2016.04.001

[6] Vanin S Utilità e limiti dell'identificazione tramite DNA delle specie di insetti di interesse forense in Italia e Europa [Utilities and limits of DNA identification of insect species of forensic interest in Italy and Europe]. Riv Ita Med Leg. 2008;30: 1403–1411. Italian.

[7] Amendt J, Campobasso CP, Gaudry E, et al. Best practice in forensic entomology—standards and guidelines. Int J Legal Med. 2007;121:90–104. 16633812 10.1007/s00414-006-0086-x

[8] Giordani G, Grzywacz A, Vanin S Characterization and identification of puparia of *Hydrotaea* Robineau-Desvoidy, 1830 (Diptera: Muscidae) from forensic and archaeological contexts. Paper presented at: 2nd International Conference in Funerary Archaeoentomology and Soldier. 2017 June 7; Treviso, Italy. 10.1093/jme/tjy142 30137441

[9] Grzywacz A, Hall MJ, Pape T, et al. Muscidae (Diptera) of forensic importance—an identification key to third instar larvae of the Western Palaearctic region and a catalogue of the muscid carrion community. Int J Legal Med. 2017;131:855–866. 27924407 10.1007/s00414-016-1495-0 PMC5388714

[10] Martín-Vega D, Hall MJR, Simonsen TJ Resolving confusion in the use of concepts and terminology in intrapuparial development studies of Cyclorrhaphous Diptera. J Med Entomol. 2016;53:1249–1251. 27524822 10.1093/jme/tjw081

[11] Skidmore P The biology of the muscidae of the world. Dordrecht (Netherlands): Springer; 1985.

[12] Altschul SF, Gish W, Miller W, et al. Basic local alignment search tool. J Mol Biol. 1990;215: 403–410. 2231712 10.1016/S0022-2836(05)80360-2

[13] Sievers F, Wilm A, Dineen D, et al. Fast, scalable generation of high-quality protein multiple sequence alignments using Clustal Omega. Mol Syst Biol. 2011;7:539. 21988835 10.1038/msb.2011.75 PMC3261699

[14] Kumar S, Stecher G, Tamura K MEGA7: Molecular Evolutionary Genetics Analysis version 7.0 for bigger datasets. Mol Biol Evol. 2016;33:1870–1874. 27004904 10.1093/molbev/msw054 PMC8210823

[15] Letunic I, Bork P Interactive tree of life (iTOL) v3: an online tool for the display and annotation of phylogenetic and other trees. Nucleic Acids Res. 2016;44:W242–W245. 27095192 10.1093/nar/gkw290 PMC4987883

[16] Grzywacz A, Wallman JF, Piwczyński M To be or not to be a valid genus: the systematic position of *Ophyra* R.-D. revised (Diptera: Muscidae). Syst Entomol. 2017;42:714–723.

[17] Lefebvre F, Pasquerault T Temperature-dependent development of *Ophyra aenescens* (Wiedemann, 1830) and *Ophyra capensis* (Wiedemann, 1818) (Diptera, Muscidae). Forensic Sci Int. 2004;139:75–79. 14687777 10.1016/j.forsciint.2003.10.014

[18] Greenberg B Flies as forensic indicators. J Med Entomol. 1991;28:565–577. 1941921 10.1093/jmedent/28.5.565

[19] Greenberg B, Kunich JC Entomology and the law: flies as forensic indicators. New York (NY): Cambridge University Press; 2005.

[20] Huchet JB L’archéo-entomologie: les insectes nécrophages associés aux soldats de Carspach. [Archaeoentomology: the necrophagous insects associated with the soldiers of Carspach]. In: Schnitzler B, Landolt M, editors. A l’est du nouveau! Archéologie de la Grande Guerre en Alsace et en Lorraine. [To east, again! Archeology of the Great War in Alsace and Lorraine]. Strasbourg (France): Musée de Strasbourg, collection Archéologie; 2013 p. 109–110. French.

[21] Greenberg B Forensic entomology: case studies. Bull Entomol Soc Am. 1985;31:25–28.

[22] Vanin S, Gherardi M, Bugelli V, et al. Insects found on a human cadaver in central Italy including the blowfly *Calliphora loewi* (Diptera, Calliphoridae), a new species of forensic interest. Forensic Sci Int. 2011;207:e30–e33. 21282022 10.1016/j.forsciint.2010.12.004

[23] Papp L Family Milichiidae In: Soós A, Papp L, editors. Catalogue of Palaearctic Diptera, vol. 10 Budapest: Akadémiai Kaidó; 1984 p. 110–118.

[24] Papp L, Wheeler TA Family Milichiidae In: Papp L, Darvas B, editors. Contributions to a manual of Palaearctic Diptera, vol. 3 Budapest: Science Herald; 1998 p. 315–324.

[25] Leto Barone G, Liotta G, Raspi A Indagini entomologiche. Metodi e scelte. Il Sarcofago dell’imperatore. Studi, ricerche e indagini sulla tomba di Federico II nella Cattedrale di Palermo. [Entomological investigations. Methods and choices. The sarcophagus of the emperor. Studies, researches and investigations on the tomb of Federico II in the Cathedral of Palermo]. Palermo (Italy): Regione Siciliana. Assessorato dei Beni Culturali ed Ambientali e della Pubblica Istruzione; 2002 p. 107–113. Italian.

[26] Braack LEO Arthropods associated with carcasscs in the northern Kruger National Park. S Afr J Wildl Res. 1986;16:91–98.

[27] Carles-Tolrá M, Díaz B, Saloña M Algunos dípteros necrófilos capturados sobre cadáveres de cerdos en el País Vasco (España) (Insecta: Diptera: Brachycera). [Some necrophilous diptera captured on pig carcasses in the Basque Country (Spain) (Insecta: Diptera: Brachycera)]. Heteropterus Rev Entomol. 2012;12:213–222. Spanish.

[28] Díaz-Aranda LM, Martín-Vega D, Gómez-Gómez A, et al. Annual variation in decomposition and insect succession at a periurban area of central Iberian Peninsula. J Forensic Leg Med. 2018;56:21–31. 29525582 10.1016/j.jflm.2018.03.005

[29] Vanin S, Tasinato P, Ducolin G, et al. Use of *Lucilia* species for forensic investigations in Southern Europe. Forensic Sci Int. 2008;177:37–41. 18079080 10.1016/j.forsciint.2007.10.006

[30] Lo Pinto S, Giordani G, Tuccia F, et al. First records of *Synthesiomyia nudiseta* (Diptera: Muscidae) from forensic cases in Italy. Forensic Sci Int. 2017;276:e1–e7. 28526458 10.1016/j.forsciint.2017.05.003

[31] Giordani G, Tuccia F, Floris I, et al. First record of *Phormia regina* (Meigen, 1826) (Diptera: Calliphoridae) from mummies at the Sant'Antonio Abate Cathedral of Castelsardo, Sardinia, Italy. Peer J. 2018;6:e4176. 29312816 10.7717/peerj.4176 PMC5756611

