# Supplementary material for: Record of Leptometopa latipes (Diptera: Milichiidae) from a human cadaver in the Mediterranean area
Source: Forensic Sci Res. 2018 Oct 9;4(4):341–7. doi: 10.1080/20961790.2018.1490473 (PMC6968644; doi:10.1080/20961790.2018.1490473)
Supplement: Supplemental Material [file TFSR_A_1490473_SM4595.docx]

**Supplementary material**

Table S1. Sequences of Milichiidae downloaded from GenBank and BOLD and included in the analysis.

| **Family** | **Species** | **NCBI** |  | **BOLD** |
| --- | --- | --- | --- | --- |
| **Milichidae** | *Leptometopa latipes* | KR755741.1  KR434028.1  KR671912.1  KP045736.1  KT619898.1 |  | ASDMT1749-11  BARSL464-16  BARSM1099-17  BARSM1282-17 |
|  | *Leptometopa halteralis* | KR968305.1  KR968352.1 |  |  |
|  | *Milichiella arcuata* | KR658784.1 |  | OPPFQ2054-17  OPPQQ542-17 |
|  | *Neophyllomyza quadricornis* | KR513493.1  KR513121.1  KR506618.1  KR505032.1  KR510674.1 | KR508282.1  KR508275.1  KR514719.1  KR457430.1 | CNWBB606-13  JWDCA911-10  JWDCA913-10  JWDCA933-10  JWDCB322-10 |
|  | *Pholeomyia indecora* | KT116944.1  KR519912.1  KR671964.1  KR664827.1  KT708664.1  KT708553.1  KT708533.1 | KT705705.1  KT705966.1  KT708247.1  KT707316.1  KT707103.1  KT706692.1  KT706604.1 |  |
|  | *Paramyia nitens* | KR520034.1  KR519577.1  KR518018.1  KR517963.1  KR640802.1  KR639809.1  KR396309.1  KR395916.1  KR395673.1  KR395622.1 | KR639590.1  KR639232.1  KR639085.1  KR637943.1  KR637942.1  KR637536.1  KR396279.1  KR396240.1  KR395535.1 |  |
|  | *Madiza glabra* | KR667790.1  KM936729.1  KR652358.1 |  |  |
|  | *Desmometopa sordida* | KR756711.1  KR972336.1  KR770933.1  KR747521.1  KR515099.1 |  |  |
| **Drosophilidae** | *Drosophila melanogaster* | KJ767244.1  KJ767243.1 |  |  |

Table S2. Published records of *L. latipes* from decomposing organic matters, animals and excrements.

| **Location** | **Context** | **Reference** |
| --- | --- | --- |
| **EUROPE** |  |  |
| Czech Republic | Decaying Pig foot | Barták & Roháček, 2011 [[1](#_ENREF_1)] |
| Hungary | poultry houses | Farkas & Papp 1989 [[2](#_ENREF_2)] |
| Italy (Lampedusa Island) | sarcophagus of Federico II | Raspi et al., 2009 [[3](#_ENREF_3)] |
| Spain | Decaying Pig | Carles Tolra et al., 2012 [[4](#_ENREF_4)] |
| **ASIA** |  |  |
| India | Fish infestation | Esser, 1988 [[5](#_ENREF_5)] |
| **AFRICA** |  |  |
| South Africa | Carcasses of freshly killed *Aepyceros melampus* | Braack, 1986 [[6](#_ENREF_6)] |
| **North AMERICA** |  |  |
| Indiana | Excrements of *Nycticeius humeralis* | Whitaker et al, 1991[[7](#_ENREF_7)] |
| New York | Nests of *Otus asio* and *Falco sparverius* | Philips & Dindal, 1990 [[8](#_ENREF_8)] |

**Supplementary references**

1. Barták M, Roháček Jc. Records of interesting flies (Diptera) attracted to meat baited pyramidal trap on sapping stump of European walnut (Juglans regia) in Central Bohemia (Czech Republic). Casopis slezského zemského muzea (A). 2011;60(3):223-233.

2. Farkas R, Papp L. Species composition and breeding sites of fly communities (Diptera) in caged-layer houses in Hungary. Parasit Hung. 1989;22:93-97.

3. Raspi A, Pisciotta S, Sajeva M. *Milichiella lacteipennis*: new record for Lampedusa Island (Italy). Bull Insectol. 2009;62(2):133-135.

4. Carles-Tolrá M, Díaz B, Saloña M. Algunos dípteros necrófilos capturados sobre cadáveres de cerdos en el País Vasco (España) (Insecta: Diptera: Brachycera). Heteropterus Rev Entomol. 2012;12:213–222.

5. Esser JR. Assessment and reduction of insect infestation of cured fish in South East Asia, with laboratory studies on *Chrysomya raegacephala* (fab.), a principal causative agent. Durham: Durham University; 1988.

6. Braack LEO. Arthropods associated with carcasscs in the northern Kruger National Park. S Afr J Wildl Res. 1986;16(3):91-98.

7. Whitaker JO, Clem P, Munsee JR. Trophic structure of the community in the guano of the evening bat N*ycticeius humeralis* in Indiana. Am. Midl. Nat. 1991;126(2):392-398.

8. Philips JR, Dindal DL. Invertebrate populations in the nests of a screech owl (*Otus asio*) and an american kestrel (*Falco sparverius*) in central New York. Entomological news. 1990;101:170-192.
